# Automating Installation of the Integrating Biology and the Bedside (i2b2) Platform

**DOI:** 10.1177/1178222618777749

**Published:** 2018-06-04

**Authors:** Kavishwar B Wagholikar, Michael Mendis, Pralav Dessai, Javier Sanz, Sindy Law, Micheal Gilson, Stephan Sanders, Mahesh Vangala, Douglas S Bell, Shawn N Murphy

**Affiliations:** 1 Massachusetts General Hospital, Boston, MA, USA; 2 Harvard Medical School, Boston, MA, USA; 3 Partners HealthCare, Boston, MA, USA; 4 University of California, Los Angeles, Los Angeles, CA, USA; 5 University of California, San Francisco, San Francisco, CA, USA; 6 UMass Medical School, Worcester, MA, USA

**Keywords:** Information Storage and Retrieval/methods, Cohort Studies*, Health Information Exchange*, Health Information Interoperability*, Data Warehousing*, Biomedical Research/organization & administration

## Abstract

Informatics for Integrating Biology and the Bedside (i2b2) is an open source clinical data analytics platform used at more than 150 institutions for querying patient data. An i2b2 installation (called hive) comprises several i2b2 cells that provide different functionalities. Given the complex architecture of i2b2 installation, creating a working installation of the platform is challenging for new users. This is despite the availability of extensive documentation for i2b2 and access to a large and active mailing list community of i2b2 users. To address this problem, we have created an automated installation package, called i2b2-quickstart, which automatically downloads the latest i2b2 source code and dependencies, and compiles and configures the i2b2 cells to create a functional i2b2 hive installation. This package will serve as a convenient starting point and reference implementation that will facilitate researchers in the installation and exploration of the i2b2 platform.

## Introduction

Informatics for Integrating Biology and the Bedside (i2b2) is an open source clinical data analytics platform used at more than 150 health care institutions for querying patient data. The platform is composed of several i2b2 cells that provide different services, and the cells communicate with each other using XML web services. However, as the platform has several components, it can take several weeks for new users to read the documentation and create a working installation of the platform. The level of effort necessary to establish a new i2b2 hive installation represents a major obstacle for wider utilization of the platform.^1^

## Background

The initial funding for i2b2 came from the National Institutes of Health. Subsequently, it has developed into an international project with many active developers coordinated by the i2b2 tranSMART foundation.^2,3^ The goal of i2b2 project is to provide clinical investigators with the software tools necessary to collect, manage, and analyze project-related clinical research data. The project provides a software suite (i2b2 platform) to construct and manage the data, and the platform has been deployed at more than 200 sites worldwide for providing cohort-querying services to clinical researchers.

The i2b2 platform is composed of multiple cells that communicate using XML web services. Each cell provides a unique service; for example, user identity management, ontology management, or natural language processing. Alongside the 5 core cells, there are optional i2b2 cells and tools for importing data,^4,5^ translation of Health Quality Measure Format (HQMF),^6^ translation to Fast Healthcare Interoperability Resources (FHIR),^7,8^ image management,^9^ federated querying, data analysis,^10^ disease-specific analytics,^11–13^ and other functionalities.^14^ This modular design facilitates the addition of new cells, and therefore new functionalities, which enable the extension of the i2b2 platform to a wide range of use, cases, and environments.^15^ The i2b2 platform is often used to replicate data from the institutional electronic health record (EHR) using a sidecar approach. The ability to integrate disparate data types with varying dimensionality into a single cohesive software infrastructure enables i2b2 to form the backbone of large-scale clinical research projects. Consequently, i2b2 has been adapted to build multi-institutional networks^16^ and forms a central component in the infrastructure of many institutions that have Clinical and Translational Science Award (CTSA) and Patient-Centered Outcomes Research Institute (PCORI) award.^17,18^

### Challenges with scientific software

In comparison with most general software packages, i2b2 is complex because it provides a generic implementation to handle a wide range of operations for data storage, querying, and user interactions. It comprises a disparate set of web services that work in unison. The i2b2 platform has been developed by researchers and is based on a previous hospital-specific implementation. Given its scientific domain, i2b2 shares several characteristics with other open source scientific software: it is developed by researchers with extensive domain expertise, engineered using an agile approach, is challenging to test due to a wide range of use cases, and is difficult to install.^19^

However, unlike most scientific software, i2b2 has been widely utilized in the production setting, and a large community of active users and developers exist for it. Institutions considering installation of i2b2 first conduct a feasibility and exploration exercise to review the online documentation, tutorials, and demonstration version of i2b2.^20^ During this process, they often seek help from the user mailing list.

### Development of new functionality and cells/web services in i2b2

Apart from the infrastructure team managing the i2b2 repository, researchers seeking to extend i2b2 to include new functionalities must also obtain a thorough understanding of the architecture and configuration settings. A working demonstration installation is therefore useful for developers attempting to integrate new functionalities in the platform. The current pathway for new developers is to use the online demonstration version to develop the proof-of-concept version of their i2b2 cell/service. Eventually, these innovators review the documentation and install the i2b2 platform on their local machine, and then proceed to integrate their code within the i2b2 source code.

### Architecture of an i2b2 hive

**Figure 1.**
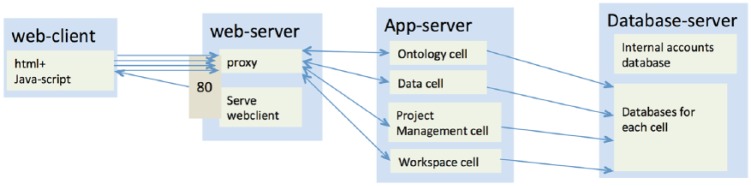
High-level view of Integrating Biology and the Bedside (i2b2) platform components.

An overview of the core i2b2 platform is presented in Figure 1 and Table 1. An i2b2 installation consists of 3 components: (1) an HTML web client (frontend), (2) Web services, and (3) an SQL database (backend). The core database is relational and includes one of PostgreSQL server, Microsoft SQL Server, or Oracle SQL server. A core i2b2 installation also includes 5 cells: (1) project management (PM) for setup of the hive, (2) ontology management (ONT) for definitions and concepts, (3) data repository (CRC) for storing and querying clinical data, (4) file repository (FR) that manages i2b2 files, and (5) workplace (WORK) that manages user-specific XML objects. The components and cells require multiple dependencies (Table 2), and finally, the web client has 2 configurations (Table 3): one for a general user and the other for administrators.

**Table 1.** Summary of the top-level components of the i2b2 platform.

Top-level component	Implementation	Summary description
Web server	Web servers like Apache or Microsoft Internet Information Server	HTML and JavaScript graphical user interface for end-users to build and run population queries
Application server	Web services on JBoss WildFly	Set of services in XML SOAP format that provides the backend to the web client. These services provide user management, authentication, and translate the user queries for execution on the SQL database
Database	PostgreSQL, Oracle, or Microsoft SQL	The database contains patient data and the user data

Abbreviation: i2b2, Integrating Biology and the Bedside.

**Table 2.** The configurations made by the program.

Configuration	Location of the configuration
WildFly data sources for each of the i2b2 cells/services	WildFly_Home eg, /opt/JBoss/WildFly/standalone/ crc-ds.xml, im-ds.xml, ont-ds.xml, pm-ds.xml,work-ds.xml Data source, IP address, port username, and password for each of the cells
Http server proxy	Web server config dir: eg, /etc/httpd/conf.d/ i2b2_proxy.conf URL prefix (including IP address and port) for proxying i2b2 web services for the web client
Http server web client and administration client	IP address and port of proxy for i2b2 web services This is the external IP address of the platform and is the only port which may be exposed to the external environment
Database: URLs of the i2b2 web services	I2b2 web service URL for the cells to discover each other
Database	Accounts for each of the web services: username and password. The accounts have access restricted only to specific tables/databases in the server

Abbreviations: i2b2, Integrating Biology and the Bedside; IP, Internet Protocol.

**Table 3.** Dependencies for installing the i2b2 platform.

I2b2 component	Dependency	Description
Compilation of source code for i2b2 web services	Apache-ant, Java jdk	Unlike maven, ant requires all the dependencies available on the file system. Ant facilitates the build java compilation
Web server	PHP5, SSL module, and proxy module	The server side of the web client is based on PHP
JBoss WildFly	Drivers for PostgreSQL, Oracle, and Microsoft SQL database	JBoss WildFly is an application server that maintains a pool of connections to the database to optimize application response times
For quickstart package	sed bzip2 tar git wget unzip patch screen	Packaging tools are required to unpack the source code and dependencies. Sed id is useful for embedding configuration parameters into template configuration files

Abbreviation: i2b2, Integrating Biology and the Bedside.

The SQL database contains patient data as well as configuration information for each of the core i2b2 cells. Specifically, the database server hosts a cell-specific database for each of the cells in the hive, and these are accessible only through the corresponding cell. Password-protected database accounts enforce the table-specific database access for the cells.

The WildFly server provides the framework to host the i2b2 web services based on enterprise Java and Apache Axis2 and facilitates the scalability of web services. Each i2b2 cell has a corresponding WildFly data source, for which WildFly maintains a pool of connections with the SQL database server. The data source configuration corresponds to the cell-specific database accounts.

The web server hosts webpages containing HTML and JavaScript for the i2b2 web client and also serves as a proxy to route the asynchronous JavaScript (Ajax) calls from the web client to web services running on the WildFly server. Accordingly, it has 2 configuration parameters: (1) the “external” Internet Protocol (IP) address of the web server and (2) the IP address and port of WildFly. The IP address of the web server is the IP address to which i2b2 users connect. This is embedded in the JavaScript of the web client. This is the only IP address and port in the i2b2 installation that needs to be exposed to the users, who will connect to the hive by using the web client. The IP address and port of the WildFly server are embedded in the proxy module configuration of the Apache web server. The use of a proxy allows the hive to be isolated from the Internet—only the proxy is exposed to the Internet, and only the proxy can communicate with the i2b2 cells/web services.

The flow of control is as follows: The user loads the web client in an Internet browser to interact with the i2b2 installation. The web client exchanges SOAP XML messages with the web services through the proxy. The web services are themselves stateless, and they retrieve information from the backend SQL database. For example, when the user requests a login, the web client passes the user credentials to the project management cell, which in turn verifies the credential in the database. Similarly, when the user requests counts of patients with a particular diagnosis, the web client invokes the data management cell, which generates and executes an SQL query on the “fact” table in the backend SQL database and returns the count to the frontend web client.

### Challenges in installing the i2b2 platform

The flexibility and extensibility that enables the application of i2b2 to so many clinical research scenarios come at the cost of simplicity, and this is particularly evident in installation of the i2b2 software.^19^ The i2b2 platform is composed of multiple components and numerous web services that must work in unison (Figure 1). Although each individual component of i2b2 is well documented, it often takes a new user several days or weeks to create a working installation of the entire platform, even with the support of online tutorials, installation tools,^1^ and an active user group. The effort required to establish a new i2b2 hive installation is therefore a major obstacle for wider utilization of the platform, especially in smaller projects with limited informatics experience.

To address this problem, we have created an automated installation package, called i2b2-quickstart, which automatically downloads the latest i2b2 source code and dependencies, and then compiles and configures the i2b2 cells to create a functional i2b2 hive installation. The quickstart package reduces the time to install i2b2 from weeks to a few minutes. This package will serve as a convenient starting point and reference implementation to facilitate researchers in the installation and exploration of the i2b2 platform.

## Methods

We developed a quickstart package to automatically download and compile the latest i2b2 source code and to install and configure the components in the Linux environment. We isolated external dependencies that may be unavailable or unsupported in the near future and hosted them in a cloud environment. In addition, we tested the performance of the quickstart package on a virtual machine in the Amazon Cloud environment using CentOS 7 as the operating system. We carried out our experiment using Amazon EC2 instance of type t2.medium, having 2 cores, 4 GB memory, and 20 GB disk space. The details of the quickstart installation are as follows.

We developed a bash program to automate the following steps:

1. Download the latest i2b2 source code, including the web services, web client, and demonstration database.
2. Download the Apache Ant and JDK dependencies and compile the web archive (war) file for web services.
3. Download and install web server and configure the proxy and JavaScript for Ajax calls.
4. Download and install JBoss WildFly server and configure the data sources for all web services.
5. Download and install PostgreSQL server.
6. Create databases for the i2b2 web services and configure database user accounts for web services.
7. Load the demonstration data into the database.
8. Run the web application and database servers.

**Figure 2.**
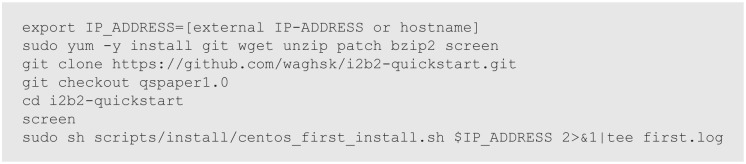
Steps to execute the quickstart package for installing the Integrating Biology and the Bedside (i2b2) platform.

**Figure 3.**
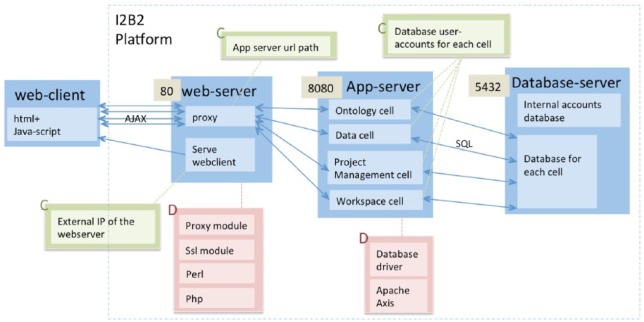
Numerous dependencies and configuration settings pose a challenge to creating a working installation of the Integrating Biology and the Bedside (i2b2) platform. Dependencies are indicated with the letter “D” (pink boxes) and configurations are indicated with the letter “C” (green boxes). The numbers indicate the input ports of the i2b2 components. IP indicates Internet Protocol.

The only input required for the package is the external IP address of the i2b2 platform. The dependencies and configuration settings that are automatically installed and configured by the program are listed in Tables 2 and 3. The package generates a log for the installation (Figures 2 and 3). The source code is available at https://github.com/waghsk/i2b2-quickstart.

## Results and Discussion

We have created the i2b2 quickstart package to automatically download, compile, and configure the i2b2 platform to run a functioning demonstration instance. In contrast to the manual installation of i2b2, our quickstart package requires a single input parameter, that is, the Internet protocol address of the i2b2 host machine. On a CentOS 7 instance in the Amazon Cloud, the quickstart package completed installation of i2b2 platform in 11 minutes. Table 4 shows the installation time distribution. Although the download and compilation of the i2b2 source code required little time, the highest proportion of time was required to load the demonstration data set into the database server.

**Table 4.** Time distribution for i2b2 installation.

Installation steps	Time required (s)
Downloading source code and dependencies	43
Compilation of web services	5
Loading demo data into the database	640

Abbreviation: i2b2, Integrating Biology and the Bedside

Rapid installation of a functional i2b2 demonstration instance will facilitate new users in exploring the platform. After the initial demonstration install, users can modify the installation to their environment: replacing the demonstration data with local data and adding local user accounts. The availability of the automated installation scripts will serve as a ready reference that augments the detailed installation documentation. This rapid installation will also be useful for infrastructure teams to efficiently instantiate new instances of i2b2 platform at different sites.

One of the primary challenges for installing i2b2 is the manual collation of dependencies. The quickstart package includes all the required dependencies: (1) The Java classes are included in the i2b2 source code, (2) Apache Ant is included in the quickstart package on a static link, and (3) additional packages are downloaded from the CentOS repository.

The second obstacle for i2b2 installation is configuration settings to connect the platform components. Quickstart provides the default values to integrate the components, as listed in Table 2. After the initial install, developers can modify these configuration settings to suit their local environment. In particular, the user and cell accounts in database tables should be modified in the production environment.

An alternative for rapidly exploring and deploying the i2b2 platform is to use a virtual machine that has the i2b2 platform preinstalled, and such virtual machines have been previously developed. However, virtual machines tend to be used like black boxes. The quickstart package is an improvement over “preinstalled” virtual machines in that it provides an explicit reference of the installation steps, and the package can be used to create virtual machines as well as physical instances.

The i2b2 community has previously used the approach of distributing precompiled jars of the i2b2 source code to circumvent the difficulty of collating the dependencies for compiling i2b2. Although this approach is convenient for upgrading existing installations, it is only partially useful for new users, who would still need to install the web, application, and database servers.

As the quickstart package can rapidly create a working i2b2 instance using the latest source code, it can be incorporated into continuous integration pipelines for automated testing of new i2b2 releases. Developers can also use this project to check whether their code is compatible with the latest i2b2 release.^21^

After initial exploration of the quickstart i2b2 installation, we recommend the following steps to use the quickstart installation in a production environment. First, modify the database password for the cell user accounts (in the database and correspondingly in the WildFly configuration). Second, migrate the database to a different server to separate the application from the database and modify the WildFly data source configuration to point to the new database host.

The i2b2 quickstart package serves as an alternative to the i2b2 installation wizard,^1,22^ which provides a semigraphical menu system to install i2b2. The quickstart package is completely automated, in downloading the required dependencies and completing the installation, which is in contrast to semiautomatic approach of the i2b2-wizard tool that requires users to select installation options from the menu and manually download some of dependencies.

### Limitations and future work

Currently, the quickstart package is limited to the Linux operating system and has only been tested on CentOS 7. Although the package is amenable to adaptation for other iterations of the Linux operating system, it would require considerable effort to port to other operating systems. However, we are exploring the adaptation of the quickstart package to other operating systems. Furthermore, we are investigating the feasibility of containerization of the i2b2 platform, as Docker containers have been reported to be particularly useful to instantiate infrastructure for scientific computing.^23,24^

We have limited the scope of the project to the PostgreSQL database, and several i2b2 sites are known to prefer other relational databases such as Oracle and Microsoft SQL. This is due to the proprietary nature of the other databases, which prohibits their distribution in open source. Nevertheless, i2b2 instances created by the quickstart package can be reconfigured to connect to proprietary databases.

## Conclusions

This article reports on our i2b2 quickstart package, which aims to facilitate a rapid installation of the i2b2 platform. Moreover, we have provided a consolidated description of the platform architecture and installation, which we anticipate will be useful for prospective new installers and developers of the platform. We hope that our efforts will significantly reduce the time and effort needed for i2b2 platform installation.
